# Toe Box Shape of Running Shoes Affects In-Shoe Foot Displacement and Deformation: A Randomized Crossover Study

**DOI:** 10.3390/bioengineering11050457

**Published:** 2024-05-03

**Authors:** Chengyuan Zhu, Yang Song, Yufan Xu, Aojie Zhu, Julien S. Baker, Wei Liu, Yaodong Gu

**Affiliations:** 1 Faculty of Sports Science, Ningbo University, Ningbo 315211, China; 2 Department of Biomedical Engineering, Faculty of Engineering, The Hong Kong Polytechnic University, Hong Kong SAR, China; 3 Faculty of Engineering, University of Szeged, 6700 Szeged, Hungary

**Keywords:** running biomechanics, footwear design, bruised toenail, injury prevention

## Abstract

Background: Long-distance running is popular but associated with a high risk of injuries, particularly toe-related injuries. Limited research has focused on preventive measures, prompting exploration into the efficacy of raised toe box running shoes. Purpose: This study aimed to investigate the effect of running shoes with raised toe boxes on preventing toe injuries caused by distance running. Methods: A randomized crossover design involved 25 male marathon runners (height: 1.70 ± 0.02 m, weight: 62.6 + 4.5 kg) wearing both raised toe box (extended by 8 mm along the vertical axis and 3 mm along the sagittal axis) and regular toe box running shoes. Ground reaction force (GRF), in-shoe displacement, and degree of toe deformation (based on the distance change between the toe and the metatarsal head) were collected. Results: Wearing raised toe box shoes resulted in a significant reduction in vertical (*p* = 0.001) and antero–posterior (*p* = 0.015) ground reaction forces during the loading phase, with a notable increase in vertical ground reaction force during the toe-off phase (*p* < 0.001). In-shoe displacement showed significant decreased movement in the forefoot medial (*p* < 0.001) and rearfoot (medial: *p* < 0.001, lateral: *p* < 0.001) and significant increased displacement in the midfoot (medial: *p* = 0.002, lateral: *p* < 0.001). Impact severity on the hallux significantly decreased (*p* < 0.001), while impact on the small toes showed no significant reduction (*p* = 0.067). Conclusions: Raised toe box running shoes offer an effective means of reducing toe injuries caused by long-distance running.

## 1. Introduction

Long-distance events, particularly half-marathon and marathon running, are increasing in popularity worldwide [1,2,3] as people strive to improve their physical fitness [4,5]. However, these endurance events also pose a heightened risk of running-related injuries [6,7], with half-marathon and marathon runners experiencing notably high injury rates, ranging from 16.7% to 79.3% [8]. These injuries can disrupt training schedules, hinder performance, and even jeopardize the runner’s well-being [9,10].

Toe injuries, such as bruised toenails or subungual hematoma, are prevalent in distance running [9,11,12,13,14,15]. These injuries often stem from the repetitive impact of long-distance running, leading to nail thickening, subungual debris accumulation, and subungual hemorrhage, which manifest as acute and painful nail discoloration, significantly impacting runners’ training and performance [12,16]. Despite this, there has been limited research on precautionary measures for toe injuries related to long-distance running, with treatments primarily relying on the experience of doctors or coaches [12]. Therefore, research into toe injuries in long-distance running holds significant practical importance.

Although there is limited long-distance running-related toe injury research, sparse clinical investigations summed up the possible reasons and prevention measures. The primary manifestation of toe injury in distance running, subungual hematoma, can also be observed in other sports such as tennis and soccer [14] and is commonly attributed to continuous friction between the toe and the shoe’s toe box [12,16]. This hypothesis can be supported by research on subungual hematoma caused by other traumas such as accidental injury caused by mechanical extrusion [17,18]. Ensuring the runner wears athletic shoes that have a bigger toe box, suitably tight shoelaces, and an efficient buffer mechanism usually seems to be a positive prevention measure to reduce the risk for subungual hemorrhage [12,13]. However, there is limited research investigating whether specific shoe designs, such as extended toe boxes, can effectively reduce the risk of toe injuries in long-distance running and what impact they may have on foot mechanics.

Improving footwear design, particularly through features like extended toe boxes, shows promise in reducing toe injuries during long-distance running [12]. However, the research on running shoes in preventing long-distance running-related injuries, to the best of our knowledge, primarily focuses on the midsole structure of the footwear [6,19], with very little attention given to the design of the shoe’s toe box. Research on these modifications can offer valuable insights into their biomechanical implications, aiding in the development of tailored injury-prevention strategies. By addressing toe injuries, runners can not only enhance their running experience but also promote long-term foot health and performance, thereby enabling more consistent training regimens and reducing the risk of being sidelined by preventable injuries. Investing in footwear research and innovation for toe injury prevention is crucial for both individual runners and the broader running community, fostering safer and more fulfilling running experiences and contributing to improved performance and long-term participation in the sport, benefiting runners of all levels.

To survey the impact between toe and toe box, the main problem is to monitor foot motion relative to the shoe. Previous studies have utilized methods involving the cutting of holes in shoe heels to monitor in-shoe foot displacement, demonstrating the feasibility of this approach, albeit primarily in the context of its correlation with athletic performance [20,21]. Therefore, cutting holes on the shoes’ upper is an efficient method to explore the foot’s movement in shoes. Additionally, the impact between toes and toe box will cause foot-shape change [7,22]. Because this impact in running commonly and observably happens in the sagittal axis, a hard impact may result in foot-shape change. Thus, changes in foot shape during running can serve as indicators of the impact forces between the toes and the toe box.

Given these circumstances, the aim of this study was twofold: (1) to develop footwear with lengthened toe boxes based on foot structural characteristics, and (2) to compare and analyze differences in foot motion and ground reaction forces (GRFs) between the modified footwear and a control group. The primary objective was to assess whether the modified footwear can potentially reduce the risk of toe injuries such as subungual hematoma through indirect measures of foot motion and biomechanics, drawing on existing research into the clinical and pathological causes of running-related injuries. A secondary objective was to examine the impact of the improved footwear on foot biomechanics. The hypothesis of this study was that improved footwear may significantly reduce biomechanical indicators associated with the risk of toe injuries caused by long-distance running. This study evaluates whether footwear with extended toe boxes can potentially reduce toe injuries caused by long-distance running, offering new preventive strategies in shoe design.

## 2. Materials and Methods

### 2.1. Footwear Design

**Figure 1:**
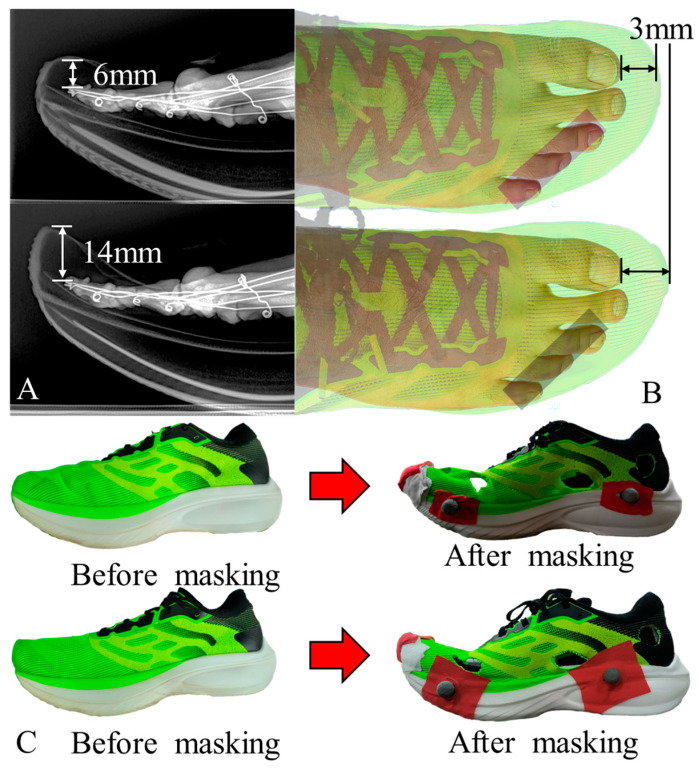
Toe box differences and masking treatment. Compared to regular toe box design, the raised toe box shoes have been extended by 8 mm along the vertical axis (**A**) and 3 mm along the sagittal axis (**B**). (**C**) Upper: Regular toe box shoes’ photo before and after masking. Lower: Raised toe box shoes’ photo before and after masking.

To reduce the impact of the toe box on the toes, we extended the toe box by 8 mm along the vertical axis and 3 mm along the sagittal axis. The extension was focused on the anterior aspect of the hallux. Due to the differences in shape between the two, in the experiment, the original running shoes were referred to as the “regular design”, while the modified ones were labeled as the “raised design”. Apart from the toe box, there were no differences in the design of the two pairs of shoes. In the experiment, the toe boxes of the shoes were masked using medical adhesive tape to wrap around the toe areas, making the lengths of the toe boxes appear nearly identical to the naked eye. This method ensured that both the participants and the experimenters conducting the study were unable to distinguish between the shoes. The specific modifications to the footwear and the masking process are depicted in Figure 1.

### 2.2. Participants

The software G∗Power 3.1.9.7 (Heinrich Heine University, Düsseldorf, Germany) was used to calculate the sample size [23]. In setting up the G*Power analysis for the two-tailed paired *t* test, α error probability was set at 0.05, the effect size was set as medium at 0.5, and the statistical power (1 − β) was set at 0.6. In this preliminary study, a lower statistical power (1 − β) was selected primarily due to cost constraints and resource limitations. This lower power increased the likelihood of Type II errors, ensuring that only strong and clear evidence could confirm the effectiveness of the shoes. Thus, this approach guaranteed that improvements were documented only when distinctly evident, maintaining the conservatism and reliability of the study outcomes. Based on the calculation’s presented settings, a minimum of 22 participants needed to be recruited.

Inclusion criteria for individuals participating in the study were as follows: (1) Below 29 years of age; (2) Shoe size 41 (European size); (3) Weekly running distance equal to or greater than 16 km; (4) No health issues, neurological or muscular disorders, or gait abnormalities; (5) No lower limb injury recorded in the past six months; (6) Half-/full-marathon best performance meeting the China Athletics Association’s elite athlete standards, i.e., half-marathon time within 1:34:00 (1.57 h)/full-marathon time within 3:24:00 (3.40 h). A total of 25 participants were eventually recruited for the experiment. Table 1 summarizes the demographic information of the participants. Before the experiment, all participants were required to sign a written consent form approved by the Institutional Ethics Committee of Ningbo University (TY2023125). This study was performed in compliance with the declaration of Helsinki.

### 2.3. Experimental Procedure

**Figure 2:**
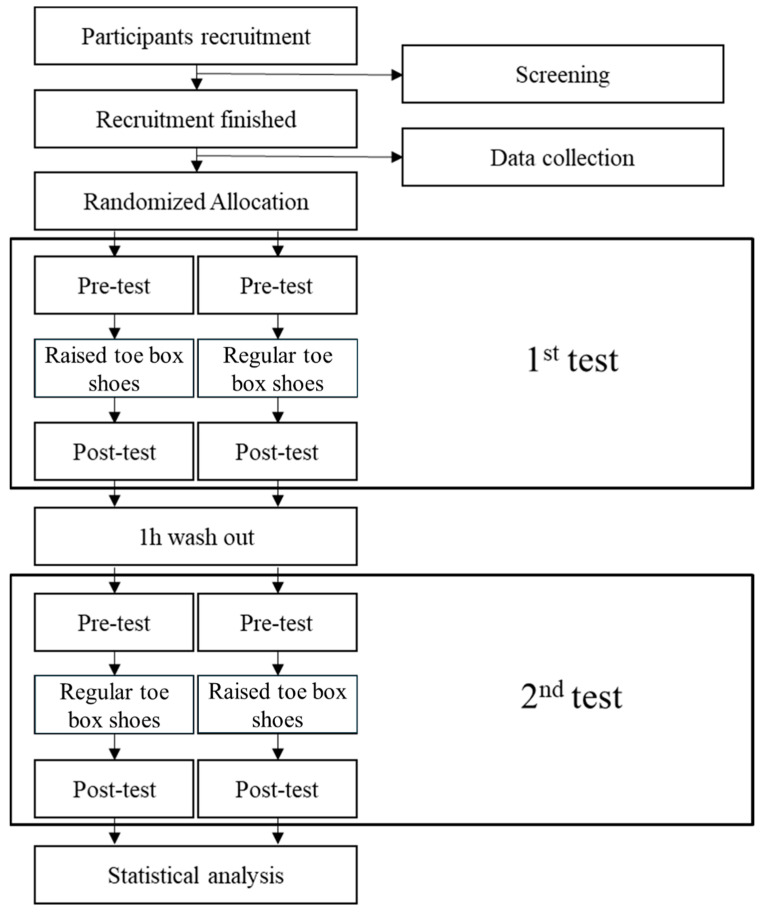
Schematic diagram of the trial.

This was a randomized crossover double-blind design trial. Each participant was asked to wear these two different toe box design shoes and finish a run at the specified speed in a laboratory setting. In the experiments, each participants’ sequences of the two tests were random. The SPSS Statistics version 27 (IBM, Armonk, NY, USA) built-in random number generator was used to generate the randomized order. Before testing, participants were required to perform a warm-up consisting of a 10-minute jog on a treadmill at a self-selected pace, ensuring they were adequately prepared for the experiment. Following the participants’ warm-up and familiarization with the experimental protocol, they wore two types of running shoes and performed running tests on a 15-meter track. Test speeds were controlled using photocells to maintain a constant velocity of 4.0 m/s with a tolerance of ±5%. The markers’ movement trajectories are captured using the VICON three-dimensional motion capture system (Vicon Metrics Ltd., Oxford, United Kingdom), with a frequency of 200 Hz. The dynamic parameters of ground reaction force (GRF) during running, including the determination of the running support phase, were collected using the Kistler three-dimensional force plate (Kistler, Winterthur, Switzerland) with a frequency of 2000 Hz. To reduce the measurement error and improve the accuracy of the data, three successful data sets were collected during each test, which was defined as the test in which the dominant leg (defined as the preferred leg during kicking) completely fell on the force plate and the participant’s running time was within the range. Each collection was separated by three minutes to avoid participant fatigue. In the test, the step point of the participant was adjusted by the experimenter to avoid the effect of the participant’s intentional adjustment of their gait on the force plate on the experimental data. The entire experimental procedure is illustrated in Figure 2.

### 2.4. Data Collection

To measure the foot’s movement in shoes, our experimental approach was based on the methodology developed by Charlotte et al.’s work [21]. Reflective markers (diameter: 10 mm) were affixed to both the bony landmarks of the foot and the midsole of the running shoes. Holes were cut to 25 mm in diameter to limit the interference from the shoe upper [24]. These include the following for the foot: the 1st metatarsal head (MH1), the 1st metatarsal base (MB1), the 5th metatarsal head (MH5), the 5th metatarsal base (MB5), the medial calcaneus (MC), and the lateral calcaneus (LC). To reduce the impact of upper openings on shoe performance [24], the placement points of MB1 and MB5 were moved 2 cm posteriorly to avoid the reinforced structure of the upper. Since this study focused on the relative displacement of the shoe and foot rather than the multi-segment model of the foot, we believe that it is feasible to move the marker point posteriorly. To study the movement of the toes in shoes, additional reflective points were attached to the 1st distal phalanx (DP1) and the 5th distal phalanx (DP5). For the running shoes, markers were placed at the anterior lateral (SAL), anterior medial (SAM), posterior lateral (SPL), and posterior medial (SPM) regions of the midsole.

Foot–shoe relative displacement is defined as the maximum relative displacement in the three-dimensional space between the foot bone marker points and shoe midsole marker points during the stance phase, including:

(1) The changing range of the distance between MH1, MH5 and the midpoint of SAL and SAM. The changing range of the distance is used to reflect the range of movement of each part of the forefoot relative to the shoe.
(2) The changing range of the distance between MB1 and the midpoints of SAM and SPM, and the changing range of the distance between MB5 and the midpoints of SAL and SPL, are used to reflect the range of movement of each part of the foot relative to the shoe.
(3) The changing range of the distance between the midpoints of MC, LC, SPL, and SPM is used to reflect the range of movement of each part of the foot relative to the shoe.

**Figure 3:**
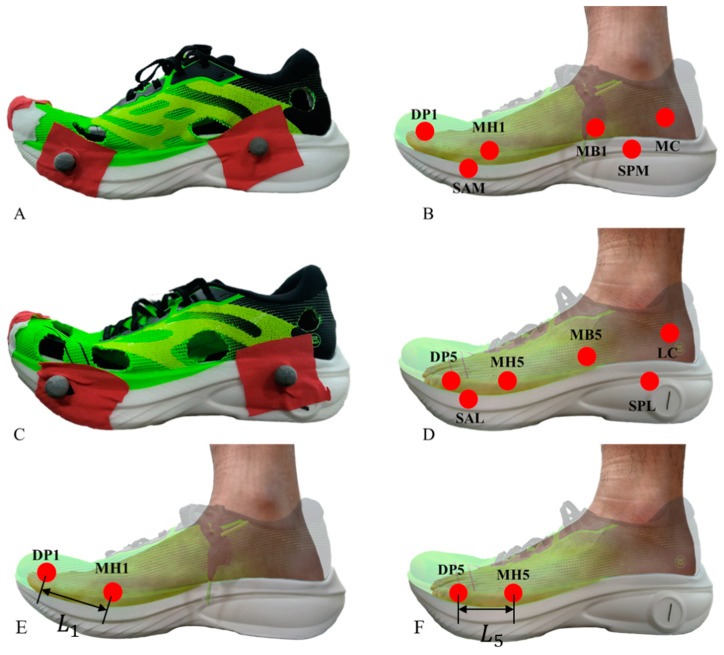
Test shoes and foot reflective marker pasting locations. (**A**) Schematic diagram of openings on the medial of shoe. (**B**) Diagram of pasting reflective points on the medial of the foot and shoe. (**C**) Schematic diagram of openings on the lateral of shoe. (**D**) Diagram of pasting reflective points on the lateral of the foot and shoe. (**E**,**F**) Calculation of the degree of toe deformation.

In addition, the degree of toe deformation, defined as the changing range of the distance between DP1 and MH1 ($L_{1}$), DP5 and MH5 ($L_{5}$), was calculated to reflect the impact severity between toes and toe box. All reflective-point pasting locations and calculate methods are presented in Figure 3. To reduce the influence of skin vibration on the experimental results, data were smoothed through a fourth-order zero-phase low-pass Butterworth filter with a cut-off frequency of 20 Hz. A fourth-order bi-directional Butterworth filter with 50 Hz cut-off frequency was applied to analogue force plate channels [21]. The vertical, antero–posterior, and medio-lateral GRF data were normalized using percentage body weight (%BW) and subsequently mapped onto the 0% to 100% range of the running support phase using custom Python code.

### 2.5. Statistical Analyses

Experimental data were analyzed using the paired samples *t*-test for differences. Normality was verified using the S–W test. If normality was violated, the Wilcoxon test was used. Ground reaction force data were analyzed comparatively using one-dimensional statistical parametric mapping (SPM1d), which explains that data variability relies on random vector field theory [25]. SPM1d statistical analysis was performed in MATLAB R2022a (The MathWorks, Natick, MA, USA), and other statistical data were analyzed using SPSS Statistics version 27, with the significance level set at 0.05. 

## 3. Results

### 3.1. Ground Reaction Force

**Figure 4:**
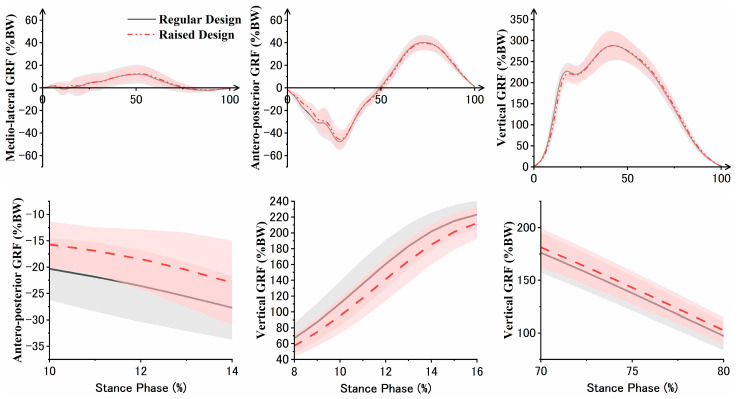
Dynamic changes in GRF. The line graph on the upper side depicts the dynamic changes in GRF during the stance phase for the participants. The SPM1d results in the box below indicate significant differences in regions marked with black boxes 1, 2, and 3 (*p* < 0.05). The line graph on the right side depicts the details corresponding to the area marked on the left-side line graph, where significant differences are indicated.

The experimental results of GRF are presented in Figure 4. Wearing raised toe box shoes led to a significant reduction and increase in vertical GRF at 8–16% (*p* = 0.001) and 58–100% (*p* < 0.001) of the stance phase, respectively. Simultaneously, antero–posterior GRF showed a significant reduction at 10–14% of the stance phase (*p* = 0.015). In this experiment, we did not observe significant changes in medio-lateral GRF.

### 3.2. Foot–Shoe Interaction and Degree of Toe Deformation

**Figure 5:**
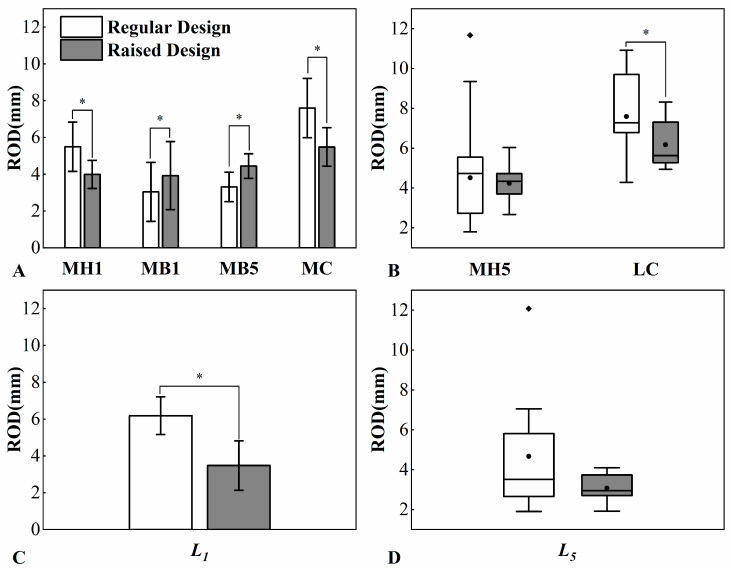
Foot–shoe relative displacement and degree of toe deformation. ROD = Range of displacement. Data for MH1, MB1, MB5, MC, and L1 are presented using bar charts, while data for MH5, LC, and L5 are represented using box plots. In the box plot, the black solid circles “●” represent the mean of the data, and the black solid diamonds ”♦” represent the outliers. The symbol ‘*’ indicates significant differences (*p* < 0.05). (**A**): Foot–shoe relative displacement results for MH1, MB1, MB5, MC; (**B**): Foot–shoe relative displacement results for MH5, LC; (**C**): Degree of toe deformation results for L1. (**D**): Degree of toe deformation results for L5.

Results of foot–shoe relative displacement and degree of toe deformation are presented in Table 2 and Figure 5. In the obtained data, MH5, LC, and $L_{5}$ did not pass the normality test (S–W test, *p* < 0.05). Therefore, comparisons were conducted using the Wilcoxon test, and the data distributions are presented as median (lower quartile, upper quartile), where upper quartile (75th percentile) and lower quartile (25th percentile) encapsulate the middle 50% of the data. Their graphical representation is shown in Figure 5 using box plots. 

When wearing a raised toe box shoe, significant reductions in relative displacements were observed for MH1, MC, and LC (MH1: raised toe box: 4.0 ± 0.8 mm, regular toe box: 5.5 ± 1.4 mm, *p* < 0.001; MC: raised toe box: 5.5 ± 1.1 mm, regular toe box: 7.6 ± 1.6 mm, *p* < 0.001; LC: raised toe box: 5.6 (5.3, 7.4) mm, regular toe box: 7.3 (6.7, 9.8) mm, *p* < 0.001). Conversely, relative displacements increased significantly for MB1 and MB5 (MB1: raised toe box: 3.9 ± 1.9 mm, regular toe box: 3.0 ± 1.6 mm, *p* = 0.002; MB5: raised toe box: 4.4 ± 0.7 mm, regular toe box: 3.3 ± 0.8 mm, *p* = 0.002). Finally, wearing shoes with a raised toe box led to a decrease in the distance change between DP1 and MH1 ($L_{1}$), suggesting a significant reduction in impact (raised toe box: 3.5 ± 1.4 mm, regular toe box: 6.2 ± 1.0 mm, *p* < 0.001).

## 4. Discussion

The primary objective was to assess whether the modified footwear can potentially reduce the risk of toe injuries such as subungual hematoma through indirect measures of foot motion and GRFs, drawing on existing research into the clinical and pathological causes of running-related injuries. A secondary objective was to examine the impact of the improved footwear on foot biomechanics. According to research objectives, we observed differences in GRF, foot–shoe relative displacement, and impact severity between wearing running shoes with a raised toe box and those with a regular toe box. Regarding GRF, wearing shoes with a raised toe box resulted in a significant decrease in vertical and antero–posterior GRFs during the loading phase and a significant increase in vertical GRF during the push-off phase. Concerning foot–shoe relative displacement, participants showed a significant decrease in the medial forefoot (MH1) and rearfoot positions (LC, MC) and a significant increase in the midfoot positions (MB1, MB5). In terms of impact severity on the toes, there was a significant decrease in the distance change between the first metatarsophalangeal joint (DP1) and the first metatarsal head (MH1), suggesting a reduced impact on the big toe. However, no significant differences were observed in the distance change between the fifth metatarsophalangeal joint (DP5) and the fifth metatarsal head (MH5). The findings of this study confirm our hypothesis that improved footwear may significantly reduce biomechanical indicators associated with the risk of toe injuries caused by long-distance running.

Regarding the changes in GRF, considering the alterations in foot–shoe relative displacement observed in this experiment, one possible explanation is that the raised toe box induced adaptive changes in the foot arch, thereby triggering these significant variations. The raised toe box may allow for better elongation of the foot arch. Greater shock absorption during ground contact may be facilitated by the greater elongation of the foot arch, resulting from a more pronounced arch drop and an increased stretching of the plantar fascia [26,27]. The significant decrease observed in both vertical and antero–posterior GRFs during the loading phase may be attributed to the raised toe box facilitating better elongation of the foot arch, enhancing its ability to absorb impacts. Subsequently, it is hypothesized that during the toe-off phase, the released elastic potential energy from the foot arch may result in a significant increase in vertical GRF. However, it should be noted that the change in GRF was very limited in this experiment. Therefore, the practical significance of this change is speculative, and further research is needed to confirm it.

The changes in foot–shoe relative displacement can also be explained by this hypothesis. As the runners’ speeds were controlled within a certain range in the experiment and served as self-controls, the energy that the foot–shoe complex needs to absorb during the loading phase is constant. Given the similarity in midsole structures of the footwear used in this experiment, the energy-absorption capacity of the footwear remains constant. When the impact is transmitted upward to the foot, the increased energy absorption by the foot arch leads to a reduction in the energy available for displacement of the forefoot and rearfoot during the loading phase. Therefore, the displacement of the forefoot and rearfoot is reduced. This change ultimately may have resulted in a decreased impact between toes and toe boxes. In the future, this hypothesis could be further investigated by directly measuring the degree of navicular drop using alternative testing methods.

The experimental results suggest a decrease in impact on the big toe, while the impact on the small toes did not significantly decrease. This may be related to the structural design of the footwear used in this experiment. The toe box of the footwear used primarily elongated over the big toe, while it remained similar in length to regular toe boxes over the small-toe positions. This design did not induce additional elongation in the lateral arch of the foot, resulting in the observed series of changes and no significant reduction in impact on the small toes. This explanation additionally addresses the skewed distribution observed in data pertaining to the lateral aspects of the foot (MH5, LC, L5), wherein the limited mobility imposed by the toe box contributes to the non-uniform data distribution. Hence, increasing the length of the raised toe box on the lateral side emerges as a potential enhancement direction for the effectiveness of this shoe modification.

Based on these results, the raised toe box design may not only have reduced the impact on the toes but also presented additional potential benefits. Firstly, the raised toe box may have triggered an increase in the elongation of the arch during the stance phase of running, offering a relatively gentle and novel approach to enhance foot core stability [28]. In addition to its relevance to sports rehabilitation [29], this could also be considered as a potential training method. By exercising the runner’s arch and inducing changes in arch stiffness, it may contribute to enhancing athletic performance [30], including the muscle power of lower limbs [31] and balance [32,33]. Furthermore, with the potential of increased energy absorption by the foot arch, a raised toe box in running shoes may potentially help reduce the load on lower limb joints, thereby lowering the risk of lower limb injuries for runners [34]. In this experiment, we observed a decrease in vertical ground reaction force during the loading phase, manifested as a reduction in loading rate and the first peak value. Although there is some controversy [35,36,37], traditionally, these changes are considered to be associated with a reduction in running injuries [38,39], potentially serving as a protective factor for runners.

The limitations of this study include: (1) the lack of direct measurement of hallux impact force; (2) variations in the probability of hallux injuries among participants of different skill levels and different gender, which cautions against overinterpreting the study’s results; and (3) the absence of further gradient-based design iterations for the footwear to identify optimal solutions. (4) The limitations of measurement methods have restricted the precision of our data acquisition. Although filtering can alleviate noise in the VICON motion capture system, artifacts from soft tissues are difficult to eliminate. Cutting holes in the shoes may alter their overall stability, potentially affecting both in-shoe and proximal joint mechanics. Subsequent investigations will concentrate on delineating the impact of footwear designs across diverse proficiency levels among runners, alongside an exploration of gradient-based variations in elongated toe box configurations, aiming to ascertain the most efficacious design parameters.

## 5. Conclusions

Our study reveals important information on the impact of a raised toe box in running shoes, demonstrating a significant reduction in degree of toe deformation and potential biomechanical changes during the loading phase. The findings suggest that this footwear modification may not only lower the risk of toe injuries but also holds promise for enhancing foot arch activity and stability. While limitations include the lack of direct measurement of hallux impact force, variations in the probability of hallux injuries among different populations, and the absence of further gradient-based design iterations for the footwear to identify optimal solutions as well as the constraints of measurement methods, these insights pave the way for future research directions. Investigating the broader effects on lower limb joints and exploring variations in running styles, especially among female runners, will contribute to a more comprehensive understanding of the implications and potential benefits of raised toe box designs in running footwear.

## Figures and Tables

**Table 1:** Information of the eligible participants.

Variable	Participants
Number	25
Age (years)	22.7 ± 3.1
Height (m)	1.70 ± 0.02
Weight (N)	613.5 ± 44.1
BMI	21.7 ± 1.6
Weekly running distance (km)	52.3 ± 22.7
Personal best (half marathon, h) ^25^	1.38 ± 0.07
Personal best (full marathon, h) ^5^	3.07 ± 0.17

Means ± SD or number as indicated. The “^25^” means 25 of the participants provided their half-marathon best score. The “^5^” means five of the participants provided their full-marathon best score. Participants’ weekly running distance and personal best score information were collected through the online questionnaire before the participant screening. Participants’ height, weight tests were performed uniformly before the experiment.

**Table 2:** Foot–shoe relative displacement and degree of toe deformation findings.

Variable Category	Location	Toe Box Design		t	*p*	95%CI
		Regular	Raised			
**Foot–shoe** **R** **elative** **Displacement**	MH1	5.5 ± 1.4	4.0 ± 0.8	6.19	<0.001	1.0~2.0
	MB1	3.0 ± 1.6	3.9 ± 1.9	−3.57	0.002	−1.4~−0.4
	MB5	3.3 ± 0.8	4.4 ± 0.7	−7.15	<0.001	−1.5~−0.8
	MC	7.6 ± 1.6	5.5 ± 1.1	6.67	<0.001	1.5~2.8
	**Location**	**Regular**	**Raised**	**Z**		***p***
	MH5	4.7(2.7, 5.6)	4.3(3.7, 4.8)	−0.12		0.916
	LC	7.3(6.7, 9.8)	5.6(5.3, 7.4)	−3.78		<0.001
**Degree of Toe Deformation**	**Location**	**Regular**	**Raised**	**t**	***p***	**95%CI**
	$L_{1}$	6.2 ± 1.0	3.5 ± 1.4	8.04	<0.001	2.0~3.4
	**Location**	**Regular**	**Raised**	**Z**		***p***
	$L_{5}$	3.5(2.7, 5.9)	3.0(2.6, 3.7)	−1.84		0.067

Comparisons for MH1, MB1, MB5, MC, and L1 were conducted using paired-sample *t*-tests, and data distribution is presented as mean ± standard deviation. MH5, LC, and L5 were compared using the Wilcoxon test, and data distribution is displayed as median (lower quartile, upper quartile).

## Data Availability

The data supporting the findings of this study can be obtained upon reasonable request from the corresponding author. However, please note that the data are not publicly available due to privacy and ethical considerations.
